# A New AS-PCR Method to Detect *CSN2*^01^ Allele, Genotyping at Ca-Sensitive Caseins *Loci* and Milk Traits Association Studies in Autochthonous Lazio Goats

**DOI:** 10.3390/ani13020239

**Published:** 2023-01-09

**Authors:** Gianfranco Cosenza, Sara Albarella, Emanuele D’Anza, Alessandra Iannuzzi, Maria Selvaggi, Mariagiulia Pugliano, Tiziana Galli, Giorgio Saralli, Francesca Ciotola, Vincenzo Peretti

**Affiliations:** 1 Department of Agriculture, University of Napoli Federico II, 80055 Portici, Italy; 2 Department of Veterinary Medicine and Animal Production, University of Naples Federico II, Via Delpino 1, 80137 Naples, Italy; 3 National Research Council (CNR), Institute of Animal Production System in Mediterranean Environment (ISPAAM), Piazzale E. Fermi, 1, 8055 Portici, Italy; 4 Department of Soil, Plant and Food Sciences, University of Bari Aldo Moro, 70126 Bari, Italy; 5 Istituto Zooprofilattico Sperimentale Lazio e Toscana “M. Aleandri” (IZSLT), UOT Lazio Sud, Str. Congiunte Destre, 04100 Latina, Italy

**Keywords:** calcium-sensitive caseins, genotyping, milk traits, autochthonous goat breeds

## Abstract

**Simple Summary:**

Identifying new alleles and mutations at calcium-sensitive casein *loci* requires continuous updating of genotyping protocols useful for association study. This study reports (1) a new, more efficient, specific PCR-based genotyping protocol to detect the *CSN2^01^* allele in goats, (2) the genetic characterization at the *CSN1S1*, *CSN2*, and *CSN1S2 loci* in three endangered goat breeds reared in the Lazio Region (Central Italy) and, (3) the association of the genotypes observed in the studied animals with parameters that might affect the milk’s traits. As regards the *CSN1S1*, *CSN2*, and *CSN1S2 loci*, no animals were found to be carriers of the *CSN1S1*^01^, *CSN1S1*^E^, *CSN2*^01^, *CSN1S2*^D^, and *CSN1S2*^0^ alleles; instead, for the *CSN1S1 locus*, a high frequency of alleles associated to a low (*CSN1S1*^F^) and high (*CSN1S1*^A*,B*^) content of the αs1 casein (αs1-Cn) content in milk, with *CSN1S1*^F^≥ *CSN1S1*^B*^ ≥ *CSN1S1*^A*^ being observed. An association between the different genotypes at the *CSN1S1 locus* and some milk traits, namely the fat and protein yielded and the fat, protein, solids-not-fat, and casein percentages without an effect on the milk yield, was observed.

**Abstract:**

Calcium-sensitive caseins are the main protein component of milk. In the goat, they are encoded by three genes (*CSN1S1*, *CSN2*, and *CSN1S2*) located on chromosome 6. A high number of alleles has been discovered for these genes in the goat species, responsible for changes in the milk’s qualitative and quantitative characteristics. This study aimed to develop an Allele-Specific PCR (AS-PCR), which allowed us to unequivocally detect goat carriers of the *CSN2^01^* allele. Subsequently, the calcium-sensitive casein *loci* genotype was investigated in three native goat breeds of the Lazio Region (Bianca Monticellana, Capestrina, and Ciociara Grigia). No individuals were carriers of the *CSN1S1*^01^, *CSN1S1*^E^, *CSN2*^01^, *CSN1S2*^D^, and *CSN1S2^0^* alleles, while a high frequency of the alleles *CSN1S1*^F^ and *CSN1S1*^A*,B*^ was observed. Association analyses between the different genotypes at the *CSN1S1 locus* and some milk traits, namely the fat and protein yielded and the fat, protein, solids-not-fat, and casein percentages without an effect on the milk yield, were observed.

## 1. Introduction

Recent achievements in molecular genetics provide the opportunity to investigate genomic regions that directly or indirectly influence animal production. Moreover, special attention is given to the enhancement and protection of native breeds of different livestock species, characterized by peculiar allele and haplotype combinations that make their production unique [1,2]. Among ruminants, the goat species shows the most significant genetic variability, especially at the *CSN1S1*, *CSN2,* and *CSN1S2 loci* that code for the three calcium-sensitive caseins: αs1-Cn, β-Cn (β casein), and αs2-Cn (αs2 casein), respectively. The *CSN1S1* gene is the most investigated and characterized by the highest level of polymorphism. The number of alleles identified at this *locus* has increased significantly over the years. To date, there are at least 22 variants associated with qualitative and quantitative differences in the content of αs1-Cn and, thus, clustered as: ‘strong’ (A, A2, A3, A′, B′, B1, B2, B3, B4, C, H, L, and M: approximately 3.5 to 4.2 g/L per allele), ‘intermediate’ (E, I, D1: ~1.1 to 1.6 g/L per allele), ‘weak’ (D, F, and G: ~0.45 to 0.6 g/L per allele), and ‘null’ alleles (01, 02, and N: 0.0 g/L per allele or trace) [2,3,4,5,6,7]. Relatively recently, two new “null” and F-like αs1-casein variants have been detected only in an indigenous Norwegian goat breed [8]. Regarding the *CSN2* gene, at least ten alleles have been identified at this *locus*: the *CSN2* alleles, such as A, A1, B, C, C1, D, E, and F alleles, which are associated with a normal (strong) amount of α-casein in goat milk (about 5–6 g/L per allele), and the *CSN2* 0 and 01 alleles, which are associated with a non-detectable amount of this protein in milk [9,10]. Recently, two new β-Cn variants, C2 and F1, less expressed than the most common, were found [11]. Studies on the *CSN1S2 locus* led to the identification of at least eight alleles related to three different quantities of αs2-Cn in milk, clustered as: normal (*CSN1S2* A, B, C, E, F, and G: ~2.5 g/L per allele), intermediate (D: ~1.5 g/L per allele), or null (0: no detectable amount of this casein) [12,13,14]. Recently, four new non-defective αs2-Cn variants named *CSN1S2* H, I, J, and K, according to the existing alphabetical order for this protein, were detected in domestic breeds and wild goat species reared in Sudan [15]. Nowadays, molecular mutations, from single nucleotide substitutions/deletions to large insertions/deletions, cause most of the above-mentioned different amounts of αs1-Cn, β-Cn, and αs2-Cn variants in the milk. They are known and quite different, and various research groups have developed quick and cheap tests for genotyping other *loci* [2,3,9,10,13,14,16,17,18]. From the first identification to today, the genetic quali-quantitative polymorphism of goat calcium-sensitive caseins has raised considerable research interest because it is related to the quality, yield, and composition of milk rather than to the technological and functional properties [19,20,21,22,23,24,25,26,27].

Therefore, the aims of the current study are (1) to set up a new efficient and specific PCR-based genotyping protocol to detect the *CSN2*^01^ allele in the goat species, (2) to investigate the distribution pattern of known variants of *CSN1S1*, *CSN2*, and *CSN1S2 loci* in Bianca Monticellana, Capestrina, and Ciociara Grigia (Figure S1), and (3) to investigate their association with the parameters that might affect the milk’s traits. Bianca Monticellana, Capestrina, and Ciociara Grigia are three endangered goat breeds reared in the Lazio Region (Central Italy). The population sizes are as follows: for the Bianca Monticellana goat, 1556 total heads, including 50 males and 1506 females; for the Capestrina goat, 739 heads, including 31 males and 708 females, and for Ciociara Grigia, 444 heads, including 24 male and 420 female goats (the data was provided by Assonapa 2022). The aptitude of these breeds is mainly milk production, intended for cheesemaking, such as Marzolina cheese, a traditional hard cylindrical-shaped cheese made exclusively from the milk of goats reared in the wild. It is eaten fresh or aged and, during the last years, has been included among Traditional Agri-food Products (TAPs) of the Lazio Region.

## 2. Materials and Methods

### 2.1. Farms and Animals

Five farms located in the Latina and Frosinone Provinces, close to each other, were included in this study. The animals were reared following the same traditional management practices of the area: the goats are left to graze in daylight hours (6/8 h/day) and return to the shed at sunset, leaving the dams with their kids at night. The kids are breastfed for up to 45 ± 5 days postpartum. Manual milking is carried out twice a day, in the morning and in the afternoon, starting from weaning to drying off (at about five months). Information concerning the parity number was also available. A total of 188 goats (125 Monticellana, 27 Capestrina, and 36 Ciociara Grigia) were used for the present study. All the animals were enrolled in the Official Birth Register (ASSONAPA) and minimally related. Blood samples were collected (19 males and 169 females) for genetic characterization. Individual milk samples (50 mL) from 169 goats (112 Bianca Monticellana, 27 Capestrina, and 30 Ciociara Grigia, respectively) at three lactation stages (60, 90, and 120-days postpartum, from May to August 2019) were collected in the morning, to evaluate the effect of casein polymorphisms on the milk yield and quality.

### 2.2. DNA Extraction

DNA was extracted from the blood by use of a Wizard DNA extraction kit (Promega–Madison, WI, USA), following the manufacturer’s instructions.

### 2.3. Genotyping at the CSN1S1 Locus

Genotyping at the *CSN1S1 locus* was carried out in stages with an initial *Xmn*I PCR-RFLP [17] to check which group of alleles the tested animals were carriers of: A*-derived alleles (A, A2, A3, A′, I, G, M, 02, H, and 01), B* alleles (B′, B1, B2, B3, C, D, D1, L, and E), and F or N alleles. DNA from subjects found to carry the A* group allele was subsequently analyzed by AS-PCR [18] to verify if they carried the 01 allele, while DNA from subjects found to carry the B* group allele was genotyped by the technique described by Jansá Perez et al. [16] to distinguish subjects in which the E allele was present.

### 2.4. Genotyping at the CSN1S2 Locus

Genotyping of the D and 0 alleles at the *CSN1S2 locus* was performed by the *Nco*I PCR-RFLP method, according to [14].

### 2.5. Genotyping at the CSN2 Locus

In order to identify carriers of the goats’ *CSN2^01^* allele at the DNA level, a new AS-PCR was set up. Sequence primers used for the AS-PCR are listed in Table S1. All primers were designed with DNASIS-Pro version 3.0 software (Hitachi, Tokyo, Japan) using the goats’ *CSN2* sequences as templates (GeneBank, nos. AJ011018, AJ011019.3). The length of the amplified fragment-spanning intron 6 (partial) to exon 7 (partial) is 463/464 bp. Amplifications were performed in a 25-μL volume containing 100 ng of genomic DNA, a 1× PCR buffer, 1.5 mM of MgCl2, 200 μM of each dNTP, 10 pmol of each primer, and 1 U of GoTaq^®^ G2 Flexi DNA Polymerase (Promega–Madison, Fitchburg, WI, USA).

The thermal conditions were: 97 °C for 2 min, 30 cycles at 94 °C for 30 s, annealing at 52.5 °C for 45 s, and extension at 72 °C for 1 min. A final extension was carried out at 72 °C for 10 min.

The amplification products were later verified by electrophoresis on a 2% agarose gel (Bio-Rad, Hercules, CA, USA) in a 0.5X TBE buffer and stained with SYBR^®^green (Lonza Rockland, Inc., Rockland, ME, USA).

### 2.6. Milk Analyses

The amount of milk (milk yield) from the morning milking was recorded on the farm. The quality parameters analyzed were: percentages of fat, protein, total casein, lactose and solids-not-fat, and amount of urea and somatic cells. Analyses were performed at the Milk Laboratory of Istituto Zooprofilattico Sperimentale Lazio e Toscana (IZSLT), the Latina section, using COMBIFOSS 6000^®^ (Foss, Hillerød, Denmark) automated equipment, consisting of:

- Milkoscan FT 6000 (the i.r. spectrophotometry method for: fat, protein, total caseins, lactose, solids-not-fat, and urea, according to the International Dairy Federation (IDF) standard 141:2013 (ISO-IDF, 2013) [28].
- Fossomatic FT 5000 (the fluoro-optoelectronic method) for somatic cells, according to the IDF 148–2:2006 method (ISO-IDF, 2006) [29].

Moreover, fat, protein, total caseins, lactose, and solid-not-fat yields were calculated.

### 2.7. Statistical Analysis

Allele frequencies were calculated by simple allele counting [30]. Possible deviations of genotypic frequencies from expectations were tested by a chi-square test to verify if the population was in the Hardy–Weinberg equilibrium. Moreover, some population genetic indices, namely gene heterozygosity (He), gene homozygosity (Ho), effective allele numbers (Ne), and the Fixation Index (FIS), were obtained by POPGENE32 software version 1.32 (PopGene: Microsoft Window-Based Freeware for Population Genetic Analysis, Edmonton, AB, Canada) [31]. The Polymorphic Information Content (PIC) was calculated according to Botstein et al. [32]. A first statistical analysis was carried out to estimate the effect of detected polymorphisms on the milk yield and composition traits of all the animals considered as a single population. In detail, a mixed model for the repeated measures [33] implemented with SAS software (SAS 9.2 Institute, Inc., Cary, NC, USA) was used to assess the possible relationship between *CSN1S1* polymorphisms and performance traits under study. Milk production data were considered as repeated measures, and the correlations between the measures in the same individual were considered in the statistical model. The statistical model included the genotype as the fixed effect (six levels), days in milk as the fixed effect of the lactation stage (3 intervals of 30 days each), the fixed effect of the breed (three levels), the fixed effect of the parity (two levels, 1st-2nd and 3rd and later), the random animal effect, and the residual error term. Subsequently, the same animals were grouped based on their genotype at the *CSN1S1 locus* into three different clusters, called strong (animals carrying A*A*, A*B*, or B*B* genotypes), intermediate (animals carrying FA* or FB* genotypes), and weak (FF goats). The model used was the same model described above. The values were considered significant at *p* < 0.05 and presented as the least squares means ± standard errors in both cases. If more than two groups were compared, a Bonferroni test was used for multiple testing.

## 3. Results

### 3.1. A New AS-PCR for the CSN2^01^ Allele Detection

**Figure 1:**
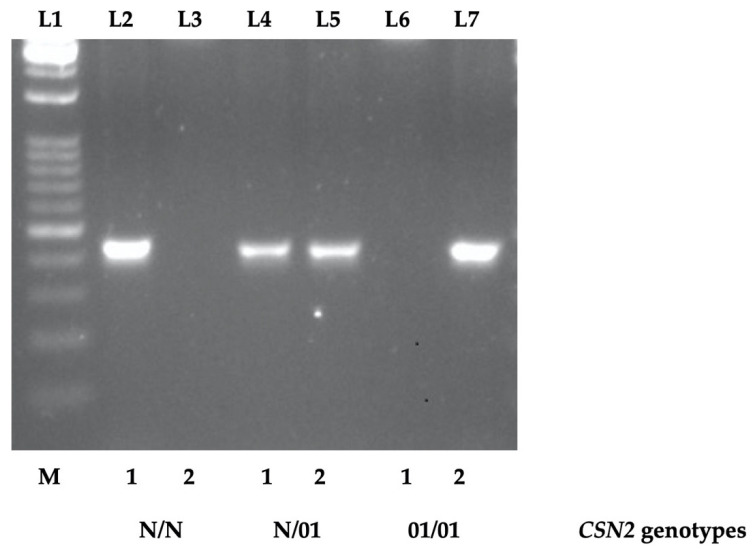
Electrophoretic patterns of fragments obtained with the new AS-PCR protocol of the goat carriers of the AJ011018:g.8915C>T mutation at the *CSN2 locus*. M = marker (1kb Opti-DNA Ladder, 0.1–10 kb, Applied Biological Materials, ABM). 1, “g.8915C” allele-specific primer (primer name, CSN2N); 2, “g.8915T” allele-specific primer (primer name, CSN201). N: non-*CSN2^01^* alleles (A, A1, B, C, C1, D, E, F, and 0); 01: *CSN2^01^* allele. N/N, N/01, and 01/01: reference samples of individuals with known genotypes. L1: Marker; L2 and L3: N/N goat; L4 and L5: N/01 goat; L6 and L7: 01/01 goat. Typing of the reference samples (2 *CSN2* N/01 and 2 *CSN2* 01/01) was accomplished eight times with identical results.

A new fast and economical method of analysis, based on AS-PCR, was set up to identify carriers of the SNP at position 373 on the seventh exon (AJ011018:g.8915C>T) that characterizes the *CSN2^01^* allele [10]. For this purpose, two different allele-specific reverse primers (named CSN2N and CSN201) that differ in the last nucleotide at the 3′-end (G→A) (Table S1) were designed. Thus, for the samples without the *CSN2^01^* allele, PCR amplification was successful only using the reverse primer with guanine at the 3′-end, whereas the *CSN2^01^* homozygote samples were successfully amplified only by the reverse primer with adenine at the 3′-end. The heterozygote samples were effectively amplified with both reverse primers (Figure 1 and Figure S2). The common forward primer (named CSN2) and the allele-specific reverse primer’s sequence are part of intron 6 and exon 7, respectively, and the amplified fragment length is 463/464 bp.

### 3.2. Genotyping

No animals were found to be carriers of the *CSN1S1^01^*, *CSN1S1^E^*, *CSN2^01^*, *CSN1S2^D^*, and *CSN1S2^0^* alleles, applying the methods already known for the *CSN1S1* and *CSN1S2* genes and the new AS-PCR protocol for *CSN2*. Table 1 shows the allele and genotype frequencies at the *CSN1S1 locus*.

Among the six allele classes investigated at the *CSN1S1 locus*, four of them were found in the studied population: A*, B*, F, and N. *CSN1S1^F^* (0.44) was the most common, and it was followed by alleles from group B* (0.30) and those from group A* (0.26). The *CSN1S1^N^* allele was present in the heterozygous state in only one subject of the Bianca Monticellana breed. This finding suggests that it is almost absent in Latium goats and, therefore, cannot be considered typical of these breeds. The application of the *CSN1S1* allele discrimination for the population variability evaluation is informative, being PIC, and calculated for the four alleles found: 0.58 in Bianca Monticellana, 0.51 in Capestrina, 0.58 in Ciociara Grigia, and 0.58 in overall the population.

### 3.3. Allele Effect on Milk Parameters

The data reported in Table 2 show the effects of the six different *CSN1S1* genotypes found in the studied population. No differences in terms of milk yield were observed. Significant differences among the genotypes were found in the fat and protein yielded and in the fat, protein, solids-not-fat, and casein contents. In detail, animals carrying the A*A* genotype produced more fat per milking if compared to the A*B* and B*F ones (*p* < 0.05). Similar results were found for the protein yield, with A*A* individuals being more productive than goats with the B*F genotype (*p* < 0.05). Moreover, A*A* and B*B* individuals produced milk with a greater fat content than their A*B* and FF counterparts. As per the protein, casein, and solids-not-fat percentage, the data show a statistically significant difference among the genotypes, with milk produced by the FF goat having a lower content of these constituents if compared with all the other genotypes.

Table 3 shows the effects of different *CSN1S1* genotypes clustered according to the αs1-Cn content on milk yield and composition. Significant differences were observed in the fat, protein, solids-not-fat, and casein percentages. The animals carrying strong genotypes produced milk with a greater percentage of fat compared to those in the weak group (*p* < 0.01). Moreover, the percentage of the protein and casein were significantly higher in milk produced by individuals with strong and intermediate genotypes if compared with the weak ones (*p* < 0.01), with the animals belonging to the strong group showing a higher value when compared with the intermediate genotypes (*p* < 0.05). Consequently, the milk produced by the strong and intermediate genotypes was richer in solids-not-fat than that produced by a goat carrying a weak genotype (*p* < 0.01).

## 4. Discussions

### 4.1. AS-PCR Protocol for CSN2^01^ Allele Detection

From the first goat milk protein polymorphisms described by Boulanger et al. [34] for αs1 and αs2-Cn and by Dall’Olio et al. [35] for β-Cn through the application of the electrophoretic technique, an increasing number of protein variants have been discovered over the years. The development of molecular genetics technologies in recent decades has also made it possible to obtain an extraordinary amount of information about the animal genome enabling the identification of causative events of the observed phenotypic differences and the identification of new alleles.

The later application of genotyping methods (PCR-RFLP, AS-PCR, ACRS-PCR, SSCP, DGGE…) has allowed a rapid and economical genotyping of individuals, regardless of the phenotypic expression, sex, and age. Identifying new alleles and mutations requires a review of existing genotyping protocols to optimize them, considering the latest findings.

From this perspective, it was necessary to set up a new AS-PCR reaction to detect carriers of the *CSN2^01^* allele in the goat. In fact, it has been observed that both allele-specific forward primers (5′- CGTGCTGTCCCTTTMTC -3′ and 5′- CGTGCTGTCCCTTTMTT -3′) proposed by Ramunno et al. [36] include, in the third-to-last nucleotide at the 3′-end, the transversion AJ011018.3:g.8913C>A responsible for the amino acid exchange, p.Ser166>Tyr, in the mature protein encoded by the most recently identified allele, *CSN2^E^* (Figure S2). This condition could reduce the efficiency and specificity of the reaction by making the genotyping method proposed by Ramunno et al. [36] ambiguous in the presence of *CSN2^E^* variant carriers. Hence, using the method developed in this study, it is now possible to quickly genotype goats at the *CSN2 locus* precisely and unequivocally.

### 4.2. Milk Traits Phenotyping

The qualitative parameters of the milk of Lazio goats, as regards the percentages of proteins, fat, lactose, not-fat-solids, and somatic cells, are perfectly aligned with those of other native and highly selected Alpine breeds [37,38]. However, considering the differences in terms of the milk yield between native and cosmopolitan breeds, the total productions per milking (the fat, protein, lactose, caseins, and solids-not-fat yields), these goats are certainly more similar to the first ones [39,40]. These findings show that the breeding of Lazio goats has its validity in consideration of the low breeding costs and that they allow the recovery of areas where other types of livestock activities would not be economically sustainable.

### 4.3. Calcium-Sensitive Caseins Loci Genotyping and Population Genetic Structure

To assess the possible correlations between the *CSN1S2* genotype and the milk parameters in the investigated goat populations, a genotyping was carried out to detect the null (0) and intermediate (D) alleles at this locus. No carriers of both the 0 and D alleles were found. This result was expected, as both of these alleles are rare and detected only in a few goat breeds. In fact, from the discovery of the 0 allele in the Napoletana goat breed [14], it was mainly observed in Italian goat breeds, such as Argentata dell’Etna [41], Maltese, Jonica [42], and Sarda [43], as well as in Saanen reared in the Bursa.Province in the Marmara Region of Turkey [44] and in some local Hungarian breeds [45]. Only in these latter populations, exceptionally, the *CSN1S2^0^* allele was observed with a relatively high incidence (0.146). Even rarer is the *CSN1S2^D^* allele, which is still identified only in Napoletana goats (0.019) [14] and Hungarian breeds (0.005) [45].

Similarly to what was observed at the *CSN1S2 locus*, the analysis of the *CSN2 locus* showed the absence of the null allele, *CSN2*^01^, in the investigated populations. This result is compatible with the ones obtained in other breeds reared in Italy and characterized by the lack or very low frequencies of this null allele [10,24,36,42,43,46,47].

In this study, no analyses have been performed to detect the null allele, *CSN2^0^*, that, similarly to the 01 allele, is characterized by a premature stop codon (codon 58) due to a single nucleotide deletion (adenine) in a row of four adenines between nt 16 and 19 of exon 7. From its identification and characterization by Persuy et al. [48] in the Pyrenean goat breed, the presence of this allele was, in fact, no longer reported in any other goat breed.

Molecular analyses showed a fair genetic variability at the *CSN1S1 locus* in the goat populations studied. Four alleles (αs1-Cn A*, B*, F, and N) and seven of the sixteen possible genotypes (Table 1) were found. In particular, this study showed the absence in these populations of alleles associated with an intermediate or absent content of this protein in the milk, with only one exception for one Bianca Monticellana goat, where we found a heterozygote for the allele N (*CSN1S1* F/N). Conversely, grouping the three genetic types, a high frequency of alleles associated with a low (*CSN1S1^F^*) and high (CSN1S1^A*,B^^*^) content of αs1 casein content in the milk, with *CSN1S1^F^* ≥ CSN1S1^B*^ ≥ CSN1S1^A*^, was observed (Table 1).

We compared the allele frequencies measured in the present work with those reported in different studies on goat populations reared in Italy and genotyped with the same or comparable techniques in this study. This comparison shows that these Lazio goat breeds have an intermediate genotype between the goat breeds reared in Northern Italy (Frisa, Orobica, Verzasca, Vallesana, Saanen, and Roccaverano), which are characterized by a high frequency of alleles associated with a null or low/intermediate αs1-Cn content. Instead, in the authocthonous breeds of Southern Italy, the alleles CSN1S1^B*^ and CSN1S1^A*^ are predominant (Table S2). The Napoletana goat breed is an exception within this panorama, and it stands out from the other breeds for the high frequency of the null allele *CSN1S1^N^* and the *CSN1S1^F^* allele (Table S2). This distinct genetic structure could be the consequence of geographic isolation after domestication. The Napoletana goat is mainly reared in the Lattari Mountains (the Campania Region), and it could have better preserved ancient alleles or variants rare or absent in other populations. On the contrary, in some breeds of Alpine origin (Saanen and Alpine Italian Chamois) the genetic selection of recent years is causing an increase in the frequency of strong and intermediate alleles at the expense of those of the weak and null [49].

For the remaining breeds, there are no genetic improvement actions. Consequently, the allelic frequencies remain somewhat stable over time, giving well-defined genetic structures to these breeds, primarily responsible for the particular chemical–physical, technological, and organoleptic characteristics of the milk produced and their derivatives.

### 4.4. Association Study

The polymorphisms of *CSN1S1* affect not only the quantity of casein in goat milk but also its structural and nutritional characteristics (the diameter of the casein micelles, calcium content, fat, fatty acid profile, and urea level) [50,51,52,53,54,55], and technological properties (the coagulation parameters, cheese yields, and organoleptic properties) [22,56]. Moreover, a greater digestibility of goat milk containing αs1-Cn weak or null content has been shown [19].

In the present study, we have found an association between different genotypes at the *CSN1S1 locus* and some milk traits, namely the fat and protein yielded and fat, protein, solids-not-fat, and casein percentages, without having an effect on the milk yield. Similar results were observed when the goats were grouped into three clusters (strong, intermediate, and weak genotypes).

This is in line with the literature being similar to those reported by [52], obtained by comparing goats with different *CSN1S1* genotypes. These authors grouped the animals into two separate clusters called high (A, B, and C alleles) and low (F, G, 0, and E alleles). They found that milk produced by the low group has lower protein and fat contents than the high group, with no difference in the milk yield and lactose concentration. Moreover, Balia et al. [51] reported a lack of association between the *CSN1S1* genotype and the milk yield in a flock of Sarda goats. Conversely, the milk protein and casein percentages were significantly affected by the genotype: the milk obtained by BB individuals was characterized by a high protein percentage than that of the AF and BF. High amounts of αs1-Cn expressed by the *CSN1S1* BB genotype have also been reported in Cilentana goats without an effect on the milk protein and total casein concentrations [27].

Interestingly, goat breeds in this report show a trend to higher protein and casein percentages in homozygote B* goats than homozygote A* ones at the *CSN1S1* gene. In support of that, Montalbano et al. [57] refer that quantifying by means of RP-HPLC B* genetic variant compared to A* show that the expression of this allele determines a higher content of αs1-casein in Girgentana goat milk.

A different degree of expression between the *CSN1S1* A* and B* alleles may be a consequence of the presence of rare and not well-characterized A* alleles associated with a lower synthesis level, such as I (intermediate) or G (low) or 02 (null) [2]. The applied genotyping method by means of *Xmn*I PCR-RFLP does not differentiate among these alleles. Another possible hypothesis is that there are individuals with B* allele variants, such as *CSN1S1*^B3^, with significant effects on the protein and casein percentages [58].

Furthermore, variations in the upstream region of the *CSN1S1* gene may affect the protein expression and significantly affects the protein percentage [59,60,61,62]. In particular, a binding site of the Activator Protein (AP-1), known to be the critical third messenger for the target genes, regulated by extracellular mediators, and involved in the gene regulation of the mammary epithelial cells, as a response to prolactin, is affected by an A→G exchange at −175 bp in the bovine *CSN1S1* promoter, associated to variations in the expression of the corresponding gene product [62]. Similarly, Ramunno et al. [7] refer that the mutation AJ504711:g653A>G seems to create an extra AP-1 binding motif in the proximal promoter sequence of the goat’s *CSN1S1* B* derived alleles. Therefore, it is possible to hypothesize that the mutation could be responsible for the observed expression level changes of goats’ strong alleles (A* e B*). Currently, studies are ongoing to verify this hypothesis (Cosenza, unpublished data).

## 5. Conclusions

The opportunity to accurately characterize the genetic structure of *loci* of interest in livestock species allows us to better plan the breeding and selection activities. The new genotyping technique reported in this paper enables a more accurate characterization of the *CSN2 locus* in goats. Future aims include the development and application of an analysis protocol for each variant at the casein loci to rate their effect on milk traits.

Moreover, in this study, the autochthonous goat breeds of the Lazio Region have been genetically characterized for the first time at the quantitative alleles of calcium-sensitive caseins. These data are essential for the correct genetic management of these breeds to avoid the modification of their population’s genetic structure and to guarantee the preservation of typical productions over time. Moreover, such breeds play two crucial functions, allowing the use of marginal areas, avoiding their abandonment, and preserving genetic variability and biodiversity.

## Figures and Tables

**Table 1:** Genotype numbers (observed and expected), allele frequencies, and population indices observed at the *CSN1S1 locus* in Capestrina, Bianca Monticellana, and Ciociara Grigia populations.

Breed		Genotype Numbers							Allele Frequency				Population Indices		
		A*A*	A*B*	B*B*	FF	B*F	A*F	FN	A*	B*	F	N	Ho	He	F_IS_
Capestrina n = 27	Obs	1	2	2	9	8	5	0	0.170	0.260	0.570	0	0.42	0.58	0.14
	Exp	0.75	2.33	1.81	8.90	8.04	5.17	0							
	χ^2^ = 0.16 *p* = 0.01 d.f. = 5														
Bianca Monticellana n = 125	Obs	15	18	9	24	35	23	1	0.284	0.284	0.428	0.004	0.61	0.66	0.06
	Exp	10.08	20.16	10.08	22.90	30.39	30.39	0.43							
	χ^2^ = 6.06 *p* = 0.01 d.f. = 6														
Ciociara Grigia n = 36	Obs	1	6	5	3	12	9	0	0.240	0.390	0.380	0	0.75	0.66	-0.14
	Exp	2.01	6.61	5.44	5.06	10.50	6.38	0							
	χ^2^ = 2.73 *p* = 0.01 d.f. = 5														
All breeds n = 188	Obs	17	26	16	36	55	37	1	0.26	0.30	0.44	0.003	0.63	0.65	0.03
	Exp	12.51	29.15	16.98	36.20	49.59	42.57	0.44							
	χ^2^ = 4.04 *p* = 0.01 d.f. = 6														

A* = A, A2, A3, A′, I, G, M, 02, H, 01; B* = B′, B1, B2, B3, C, D, D1, L, E.

**Table 2:** Effect of different genotypes at the *CSN1S1 locus* on milk yield and composition of all the animals studied.

Parameters	Genotypes					
	A*A* n = 16	A*B* n = 24	B*B* *n* = 15	A*F *n* = 34	B*F *n* = 44	FF *n* = 35
Milk yield (g/milking)	608.13 ± 66.26	521.54 ± 51.98	507.14 ± 70.83	534.46 ± 43.58	470.00 ± 39.51	552.57 ± 44.80
Fat yield (g)	29.54 ± 3.05 ^a^	21.23 ± 2.39 ^b^	25.10 ± 3.26	22.55 ± 2.01	19.96 ± 1.82^b^	22.41 ± 2.06
Protein yield (g)	22.28 ± 2.27 ^a^	18.83 ± 1.78	18.91 ± 2.42	18.80 ± 1.49	16.46 ± 1.35^b^	17.42 ± 1.53
Lactose yield (g)	26.68 ± 3.00	22.93 ± 2.35	22.26 ± 3.20	23.35 ± 1.97	20.71 ± 1.79	24.49 ± 2.03
Solids-not-fat (g)	53.08 ± 5.64	45.21 ± 4.42	44.49 ± 6.03	45.85 ± 3.71	40.26 ± 3.36	45.46 ± 3.81
Casein (g)	16.78 ± 1.67	14.10 ± 1.33	14.25 ± 1.81	14.02 ± 1.11	12.30 ± 1.01	12.77 ± 1.14
Fat (%)	4.90 ± 0.26 ^a^	4.06 ± 0.20 ^b^	4.97 ± 0.26 ^a^	4.33 ± 0.17	4.32 ± 0.15	4.08 ± 0.17 ^b^
Protein (%)	3.67 ± 0.09 ^A^	3.65 ± 0.07 ^A^	3.78 ± 0.09 ^A^	3.56 ± 0.06^A^	3.50 ± 0.05 ^a^	3.22 ± 0.06 ^B,b^
Lactose (%)	4.38 ± 0.06	4.35 ± 0.05	4.40 ± 0.06	4.33 ± 0.04	4.44 ± 0.03	4.43 ± 0.04
Solids-not-fat (%)	8.73 ± 0.10 ^a^	8.66 ± 0.08 ^a^	8.85 ± 0.10 ^A^	8.60 ± 0.07 ^a^	8.61 ± 0.06 ^a^	8.31 ± 0.07 ^B,b^
Casein (%)	2.76 ± 0.08 ^A^	2.72 ± 0.06 ^A^	2.86 ± 0.08 ^A^	2.66 ± 0.05 ^A^	2.62 ± 0.05 ^A^	2.38 ± 0.05 ^B^
Urea (mg/100 mL)	48.94 ± 2.14	49.36 ± 1.68	47.46 ± 2.14	50.05 ± 1.40	51.63 ± 1.25	50.53 ± 1.39
Somatic cell count (×10^3^)	1909.13 ± 689.73	1616.04 ± 541.07	1602.31 ± 689.73	2006.89 ± 466.35	1610.36 ± 402.43	1598.94 ± 447.55

A, B = *p* < 0.01; a, b = *p* < 0.05; A* = A, A2, A3, A′, I, G, M, 02, H, 01; B* = B′, B1, B2, B3, C, D, D1, L, E.

**Table 3:** Effect of genotypes at the *CSN1S1 locus* clustered as strong, intermediate, and weak on the milk yield and composition of all the animals studied.

	Cluster		
Parameters	Strong *n* = 55	Intermediate *n* = 78	Weak *n* = 35
Milk yield (g/milking)	542.68 ± 35.37	499.09 ± 29.23	552.57 ± 44.74
Fat yield (g)	24.57 ± 1.64	21.13 ± 1.36	22.41 ± 2.08
Protein yield (g)	19.83 ± 1.21	17.51 ± 1.00	17.42 ± 1.53
Lactose yield (g)	23.84 ± 1.60	21.90 ± 1.32	24.49 ± 2.02
Solids-not-fat (g)	47.28 ± 3.01	42.78 ± 2.49	45.46 ± 3.81
Casein (g)	14.90 ± 0.90	13.08 ± 0.75	12.77 ± 1.14
Fat (%)	4.54 ± 0.14 ^A^	4.32 ± 0.12	4.08 ± 0.17 ^B^
Protein (%)	3.69 ± 0.05 ^A,a^	3.53 ± 0.07 ^A,b^	3.22 ± 0.09 ^B^
Lactose (%)	4.37 ± 0.03	4.39 ± 0.03	4.43 ± 0.04
Solids-not-fat (%)	8.73 ± 0.05 ^A^	8.61 ± 0.05 ^A^	8.31 ± 0.07 ^B^
Casein (%)	2.77 ± 0.04 ^A,a^	2.64 ± 0.03 ^A,b^	2.38 ± 0.05 ^B^
Urea (mg/100 mL)	48.72 ± 1.12	50.94 ± 0.93	50.53 ± 1.38
Somatic cell count (×10^3^)	1693.10 ± 359.72	1779.61 ± 302.53	1598.94 ± 444.41

A, B = *p* < 0.01; a, b = *p* < 0.05. Strong genotypes = A*A*, A*B*, B*B*; intermediate genotypes = A*F, B*F; weak genotype = FF.

## Data Availability

The data presented in this study are available on request from the corresponding author.

## Supplementary Materials

The following supporting information can be downloaded at: https://www.mdpi.com/article/10.3390/ani13020239/s1, Figure S1: (a) flock of the Bianca Monticellana goat breed that goes to pasture; (b) the Capestrina goat at recovery; (c) the Ciociara Grigia goat breed at the pasture; Figure S2: Goat *CSN2* exon 7 nucleotide sequence (capital letters), plus the parzial intron flanking region (small letters). Primer sequences for AS-PCR reported by Ramunno et al. [36] are double underlined. Amino acid sequence is in uppercase and bold letters. Primer sequences for AS-PCR reported in this work are boxed. The transversion AJ011018.3:g.8913C>T responsible for the amino acid exchange, p.Ser166>Tyr, in the mature protein encoded by the *CSN2^E^* allele is highlighted in gray. The *CSN2^01^* premature stop codon is symbolized by *; Table S1: Primer sequences and position used for AS-PCR; Table S2: Comparison of the allele frequencies for the *CSN1S1 locus* in different Italian goat breeds. References [63,64] are cited in the supplementary materials.
