# Supplementary material for: A New AS-PCR Method to Detect CSN201 Allele, Genotyping at Ca-Sensitive Caseins Loci and Milk Traits Association Studies in Autochthonous Lazio Goats
Source: Animals (Basel). 2023 Jan 9;13(2):239. doi: 10.3390/ani13020239 (PMC9854881; doi:10.3390/ani13020239)
Supplement: Supplementary file 1 [file animals-13-00239-s001.zip › animals-2023764-supplementary.pdf]

Table S1. Primer sequences and position used for AS-PCR.

***CSN2*-Amplified Fragment**	**Position**	**Primer Sequence (5′-3′)**	**Primer Name**
Partial intron 6 - partial exon 7	8470-8495 *	F: AGGAGGATAATTCATCATGAATAACA	CSN2
	Complementary to 8914 - 8932 **	R: CAGGCAGAACTTTGGGCTA	CSN201
	Complementary to 8915- 8932 *	R: AGGCAGAACTTTGGGCTG	CSN2N

* Numbering of primers was according to the goat *CSN2* sequence (AJ011018.3)
** Numbering of primer is according to the goat *CSN2* sequence (AJ011019.3)

Table S2. Comparison of the allele frequencies for the *CSN1S1 locus* in different Italian goat breeds.

**Goat Breed**	***CSN1S1***						**References**
	F	N	B*	A*	E	01	
Capestrina	0.5700	-[1]	0.2600	0.170	-	-	Present work
Bianca Monticellana	0.4260	0.004	0.2800	0.2800	-	-	Present work
Ciociara	0.3800	-	0.3900	0.2400	-	-	Present work
Napoletana	0.3680	0.227	0.1800	0.1420	0.0830	-	[2]
Saanen	0.1400	-	0.0600	0.3500	0.4300	0.0200	[49]
Camosciata Alpina	0.1100	-	0.1500	0.5600	0.1800	-	[49]
Girgentana	0.2900	0.047	0.0650	0.5900	0.008	-	[63]
Sarda	0.2420	0.006	0.5200	0.2020	0.0300	0.0010	[25]
Argentata dell'Etna	0.3690	-	0.6310		-	-	[41]
Rossa Mediterranea	0.480	-	0.5200		-	-	[64]
Vallesana	0.3860	-	0.1270	0.0300	0.2830	0.1750	[42]
Roccaverano	0.3770	0.0190	0.1170	0.2270	0.2140	0.0450	[42]
Jonica	0.2820	-	0.3050	0.3500	0.0640	-	[42]
Garganica	0.2281	0.0088	0.2807	0.4298	0.0175	0.0351	[56]
Maltese	0.3710	-	0.1570	0.4140	0.0570	-	[42]
Frisa	0.5570	-	0.0070	0.1290	0.2000	0.1070	[46]
Orobica	0.9620	-	0.0080	-	0.0080	0.0230	[46]
Verzasca	0.7540	-	0.0370	-	0.2010	0.0070	[46]

[1]-: allele frequency = 0.

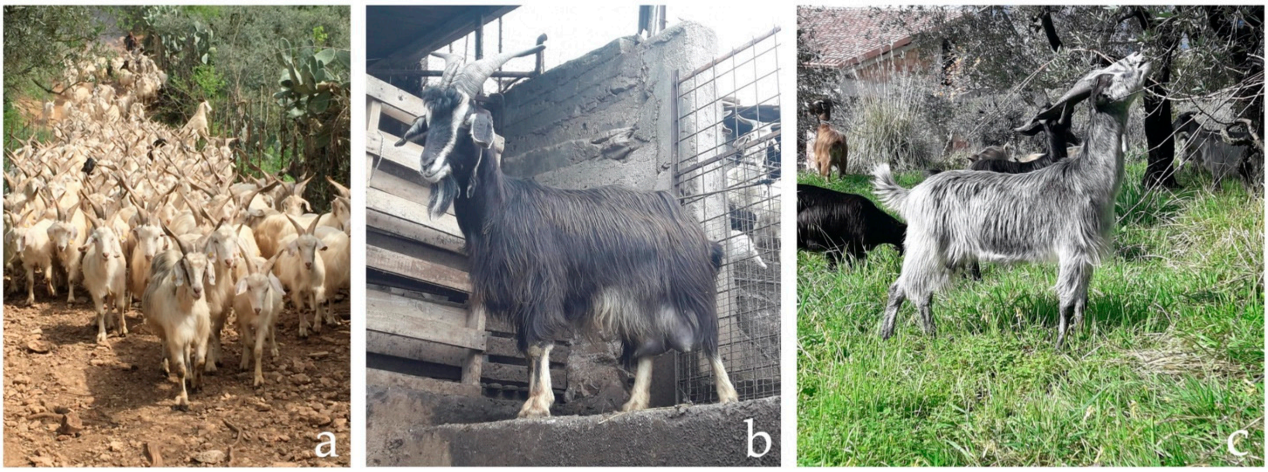

Figure S1. (**a**) flock of Bianca Monticellana goat breed that goes to pasture; (**b**) Capestrina goat at the recovery; (**c**) Cicciara Grigia goat breen at the pasture.

```
     aggaggataattcatcatgaataacaattataactggattatggactcaaagatttttttccttctttccag

 D  E  L  Q  D  K  I  H  P  F  A  Q  A  Q  S  L  V  Y  P  F  T  G  P  I  P  N  S
GATGAACTCCAGGATAAAATCCACCCCTTTGCCCAGGCACAGTCTCTAGTCTATCCCTTCACTGGGCCCATCCCTAACAG

  L  P  Q  N  I  L  P  L  T  Q  T  P  V  V  V  P  P  F  L  Q  P  E  I  M  G  V
CCTCCCACAAAACATCCTGCCTCTTACTCAAACCCCTGTGGTGGTGCCGCCTTTCCTTCAGCCTGAAATAATGGGAGTCC

P  K  V  K  E  T  M  V  P  K  H  K  E  M  P  F  P  K  Y  P  V  E  P  F  T  E  S
CCAAAGTGAAGGAGACTATGGTTCCTAAGCACAAAGAAATGCCCTTCCCTAAATATCCAGTTGAGCCCTTTACTGAAAGC

 Q  S  L  T  L  T  D  V  E  K  L  H  L  P  L  P  L  V  Q  S  W  M  H  Q  P  P  Q
CAGAGCCTGACTCTCACTGATGTTGAAAAGCTGCACCTTCCTCTGCCTCTGGTCCAGTCTTGGATGCACCAGCCTCCCCA
                                                    166
  P  L  S  P  T  V  M  F  P  P  Q  S  V  L  S  L  S  Q  P  K  V  L  P  V  P  Q
GCCTCTTTCTCCAACCGTCATGTTTCCTCCTCAGTCCGTGCTGTCCCTTTCTCAGCCCAAAGTTCTGCCTGTTCCCCAGA
                                                    A T
                                                    Y  *
K  A  V  P  Q  R  D  M  P  I  Q  A  F  L  L  Y  Q  E  P  V  L  G  P  V  R  G  P
AAGCAGTGCCCCAGAGAGATATGCCCATCCAGGCCTTTCTGCTGTACCAGGAGCCTGTACTTGGTCCTGTCCGGGGACCC

 F  P  I  L
TTCCCTATTCTTgtaagtctaaatttactaactgtgctgtttaacttctgatgtttgtatgatatttgagtaattaagag

ccctacaaaaaatcaataatgaatggttccaaaataagcatagctgagattaatgattctcagcattagttataaataga

ataagctggaaaac
```

Figure S2. The goat *CSN2* exon 7 nucleotide sequence (capital letters) plus the parzial intron flanking region (small letters). Primer sequences for AS-PCR reported by Ramunno *et al*. (1995) are double underlined. Amino acid sequence is in uppercase and bold letters. Primer sequences for AS-PCR reported in this work are boxed. Transversion AJ011018.3:g.8913C>T responsible for the amino acid exchange p.Ser166>Tyr in the mature protein encoded by *CSN2*[E] allele is highlighted in gray. *CSN2*[01] premature stop codon is symbolized by *.

References

2. Cosenza, G.; Pauciullo, A.; Gallo, D.; Colimoro, L.; D'Avino, A.; Mancusi, A.; Ramunno, L. Genotyping at the CSN1S1 locus by PCR-RFLP and AS-PCR in a Neapolitan Goat Population. *Small Rumin. Res.* **2008**, *74*, 84–90. https://doi.org/10.1016/j.smallrumres.2007.03.010.
25. Vacca, G.M.; Dettori, M.L.; Piras, G.; Manca, F.; Paschino, P.; Pazzola, M. Goat casein genotypes are associated with milk production traits in the Sarda breed. *Anim. Genet.* **2014**, *45*, 723–731. https://doi.org/10.1111/age.12188.
41. Marletta, D.; Bordonaro, S.; Guastella, A.M.; Criscione, A.; D'Urso, G. Genetic polymorphism of the calcium sensitive caseins in Sicilian Girgentana and Argentata dell'Etna goat breeds. *Small Rumin. Res.* **2005**, *57*, 133–139. https://doi.org/10.1016/j.smallrumres.2004.06.019.
42. Sacchi, P.; Chessa, S.; Budelli, E.; Bolla, P.; Ceriotti, G.; Soglia, D.; Rasero, R.; Cauvin, E.; Caroli, A. Casein haplotype structure in five Italian goat breeds. *J. Dairy Sci.* **2005**, *88*, 1561–1568. https://doi.org/10.3168/jds.S0022-0302(05)72825-3.
46. Caroli, A.; Chiatti, F.; Chessa, S.; Rignanese, D.; Bolla, P.; Pagnacco, G. Focusing on the goat casein complex. *J. Dairy Sci*. **2006**, *89*, 3178–3187. https://doi.org/10.3168/jds.S0022-0302(06)72592-9.
49. Frattini, S., Nicoloso, L.; Coizet, B.; Chessa, S.; Rapetti, L.; Pagnacco, G., Crepaldi, P. Short communication: The unusual genetic trend of αS1-casein in Alpine and Saanen breeds. *J. Dairy Sci.* **2014**, *97*, 7975–7979. https://doi.org/10.3168/jds.2014-7780.
56. Santillo, A.; Ciliberti, M.G.; D'Angelo, F.; Albenzio, M. The Effect of Alpha s1 Genotype on Some Physiological and Chemical Milk Characteristics in Garganica Goat. *Anim. Front.* **2022**, *3*, 897172. https://doi.org/10.3389/fanim.2022.897172.
63. Mastrangelo, S.; Sardina, M.T., Tolone, M.; Portolano, B. Genetic polymorphism at the CSN1S1 gene in Girgentana dairy goat breed. *Anim. Prod. Sci.* **2013**, *53*, 403–406. https://doi.org/10.1071/AN12242.
64. Guastella, A.M.; Criscione, A.; Zuccaro, A.; Tidona, F.; Marietta, D.; Bordonaro, S. Genetic polymorphism of CSN1S1 and CSN2 loci in Rossa Mediterranea goat population. *Ital. J. Anim. Sci.* **2009**, *8*, 101–103. https://doi.org/10.4081/ijas.2009.s3.101.
